# Actin Microfilament Mediates Osteoblast Cbfa1 Responsiveness to BMP2 under Simulated Microgravity

**DOI:** 10.1371/journal.pone.0063661

**Published:** 2013-05-10

**Authors:** Zhongquan Dai, Feng Wu, Jian Chen, Hongjie Xu, Honghui Wang, Feima Guo, Yingjun Tan, Bai Ding, Jinfu Wang, Yumin Wan, Yinghui Li

**Affiliations:** 1 Faculty of Aerospace Medicine, Fourth Military Medical University, Xi’an, China; 2 State Key Laboratory of Space Medicine Fundamentals and Application, China Astronaut Research and Training Center, Beijing, China; 3 Institute of Cell and Development Biology, College of Life Sciences, Zhejiang University, Hangzhou, China; St. Jude Children’s Hospital, United States of America

## Abstract

Microgravity decreases osteoblastic activity, induces actin microfilament disruption and inhibits the responsiveness of osteoblast to cytokines, but the mechanisms remains enigmatic. The F-actin cytoskeleton has previously been implicated in manifold changes of cell shape, function and signaling observed under microgravity. Here we investigate the involvement of microfilament in mediating the effects of microgravity and BMP2 induction on Cbfa1 activity. For this purpose we constructed a fluorescent reporter cell line (OSE-MG63) of Cbfa1 activity by stably transfecting MG63 cells with a reporter consisting of six tandem copies of OSE2 and a minimal mOG2 promoter upstream of enhanced green fluorescent protein (EGFP). The fluorescence intensity of OSE-MG63 showed responsiveness to bone-related cytokines (IGF-I, vitamin D3 and BMP2) and presented an accordant tendency with alkaline phosphatase (ALP) activity. Using OSE-MG63 reporter fluorescence, we performed a semi-quantitative analysis of Cbfa1 activity after treatment with simulated microgravity, microfilament-disrupting agent (cytochalasin B, CB), microfilament-stabilizing agent (Jasplakinolide, JAS) or any combination thereof. In parallel, ALP activity, DNA binding activity of Cbfa1 to OSE2 (ChIP), F-actin structure (immunofluorescence) and EGFP mRNA expression (RT-qPCR) were analyzed. Simulated microgravity inhibited Cbfa1 activity, affected the responsiveness of Cbfa1 to cytokine BMP2, and caused a thinning and dispersed distribution of microfilament. Under normal gravity, CB significantly attenuated BMP2 induction to Cbfa1 activity as well as DNA binding activity of Cbfa1 to OSE2. The addition of JAS reversed the inhibitory effects of microgravity on the responsiveness of Cbfa1 to BMP2. Our study demonstrates that disrupting the microfilament organization by CB or simulated microgravity attenuates the responsiveness of Cbfa1 to BMP2. A stabilization of the microfilament organization by JAS reverses this inhibition. Taken together, these results suggest that actin microfilament participates in BMP2’s induction to Cbfa1 activity and that their disruption might be an important contributor to microgravity’s inhibition on BMP2’s osteogenic induction.

## Introduction

During spaceflight, 1–2% of bone mass, particularly of weight-bearing bone, is lost each month [1]. The reduction of bone formation is considered to be the main cause of decrease in bone density during spaceflight [2]. Real and simulated microgravity by clinorotation inhibits the differentiation of osteoprogenitor cells into mature osteoblasts [3]–[6] and simulated microgravity by hindlimb unloading decreases the osteogenic potential of bone marrow mesenchymal stem cells (BMSCs) [7]. Taken together, bone loss induced by microgravity has been attributed to osteoblasts due to their (a) reduced proliferation and activity, (b) reduced differentiation and (c) decreased responsiveness of osteoblast to bone related factors in the microenvironment. However, the mechanisms are not fully understood [8], [9].

Microenvironmental influences such as mechanical stress and pulsed electromagnetic fields affect bone morphogenetic protein 2 (BMP2) expression and its functions during osteoblast differentiation [10], [11]. Under physiological conditions, BMP2 is a major osteogenic factor which promotes osteoblast differentiation and bone formation by increased expression of bone matrix proteins [12], [13]. BMP2 activates R-smad and kinase signaling cascades such as PI3K/Akt and MAPK, leading to activation of osteogenic transcription factors such as Cbfa1, Osx, and Msx2 [14], [15]. BMP2 also promotes migration and adhesion of osteoblasts during osteogenesis in bone regeneration [13], [16]. These effects change under microgravity. Fu and Cao demonstrated that simulated microgravity gradually decreases BMP2 mRNA levels during hindlimb suspension [17]–[19]. Under simulated microgravity, the induction effects of BMP2 on osteoblast differentiation are reduced [20], which may be caused by a reduction of MAPK signaling pathway component MEK1 [21]. The combined effects of BMP2, FGF2 and SB203580 (a p38MAPK inhibitor) significantly reverses the effects of simulated microgravity on the osteogenic differentiation of hMSCs, but not alone treatment [22], which demonstrates that microgravity affects osteogenic differentiation through a number of signaling pathways. However it is not well understood how microgravity inhibits the osteogenic actions of BMP2.

The dynamic alteration of the cytoskeleton organization induced by various stimulation such as fluid flow contributed to the modification of intracellular signals that control the differentiation, function and gene expression of osteoblasts or chondrocytes [23], [24]. In addition to activating several signaling pathways, BMP2 also induces a rapid and significant actin-microfilament cytoskeleton rearrangement during osteogenic induction, which may affect the migration and adhesion of osteoblast [16], [25], [26]. It has also been shown that collagen/integrin signaling interacts with BMP signaling to fully induce osteoblast differentiation [27]. As part of the extensive cytoskeletal system and an important microgravity sensitive sensor [28], [29], integrins αvβ play a critical role in BMP2 function on osteoblasts [30], [31]. These reports suggest that the actin cytoskeleton network plays an important role during BMP2-induced osteoblast differentiation [32]. Ours and other investigators’ work have shown that osteoblast microfilament network is disrupted under real or simulated microgravity [33]–[35], but strengthened under hypergravity [36].

Cbfa1, an important transcription factor, is essential for osteoblast differentiation and thus for skeletal morphogenesis [37], [38]. As a scaffold protein for nucleic acids, Cbfa1 is stable during cell division, remains associated with chromosomes during mitosis [39], and plays a central role in integration, organization and combinatorial assembly of DNA and its regulatory factors within the three-dimensional context of nuclear architecture [40]. It is of note that some cytoskeleton binding proteins such as Filamin B can regulate Cbfa1 activity and expression at least in part through the Smad3 pathway [41]. Smad3 is a downstream component of BMP2 signaling and can directly interact with Cbfa1 [42]. We hypothesize that actin microfilament is involved in Cbfa1 activity induced by BMP2 and participate the microgravity inhibition on Cbfa1 activity.

In this work, we establish an osteoblast reporter line to visualize Cbfa1 activity in micro- and hyper- gravity conditions in the presence of activators or inhibitors of the actin cytoskeleton.

## Materials and Methods

### Vector Construction

Using previously published techniques [43], [44], the following two partially complementary (in bold) oligonucleotides containing 6 tandem repeats of the osteoblastic specific element 2 (OSE2) were synthesized by Shanghai Xuguan Biotechnology: P1∶5-ggtaccattaatacgtaagatctccaaccacaccaaccacaccaaccacaccaaccacaccaaccacaccaaccaca**GCCG ATATAAATGCTACTGG**-3; P2∶5-cgggatccgacttgtctgttctgcaccctccagcat**CCAGTAGCATT TATATCGGC**-3. The oligonucleotides were annealed, extended by Taq polymerase, then inserted into the T-vector and named pUC57-6OSE2. A 143 bp AseI-BamHI fragment from a sequenced pUC57-6OSE2 vector was inserted into a pEGFP-N1 vector whose CMV promoter had been removed by Ase I and Bgl II. The construct was identified by limited enzyme digestion, sequenced and named p6OSE2-EGFP.

### Stable Transfection

MG63 cells which originally came from ATCC (Number: CRL-1427) were cultured in MEM medium supplemented with 10% FBS, 100 U/ml penicillin, 100 µg/ml streptomycin and 25 mmol/L Hepes in a humidified atmosphere of 5% CO_2_ and 95% air at 37°C. Two hours before transfection, media from cells in a six-well plate at 50–80% confluence was replaced with Opti-MEM (Invitrogen, USA). Cells were transfected with p6OSE2-EGFP using lipofectamine2000 (Invitrogen, USA) according to manufacturer’s instructions. About 48 h later the transfected cells were passaged using a 1∶10 split with 600 µg/ml G418 selection medium. After culturing with selective medium for 2 weeks, 6 positive clones were subcloned by limiting dilution. One of the clone was used to the following experiment after identification. The stably transfected cells were examined under a fluorescence microscope (Leica, Germany) with an H3 blue excitation filter system (420–490 nm), then expanded in medium containing G418 (500 µg/ml). The stability of fluorescence was confirmed by cryopreservation and revitalization for several times.

### Cell Treatments

OSE-MG63 cells were detached by trypsin (1∶250, Gibco) and plated at 0.5–1×10^5^/ml on glass coverslips in six-well plates. After adherence on the coverslip (about 24 h), cells were treated with different concentrations of 1.25(OH)_2_ Vitamin D_3_ (VD_3_) (0.1, 0.2, 0.4 µmol/L), IGF-I (50, 100, 200 ng/mL), BMP2 (50, 100, 200 ng/ml), CB (0.5, 2.0, 4.0 µmol/L) or JAS (12.5 nmol/L) or combination (BMP2+CB, BMP2+JAS) thereof. Fluorescence intensity and ChIP analysis were performed 48 h later. For simulated microgravity treatment, the coverslips were transferred to a biocompatible polyethylene culture box, filled with medium with or without BMP2, JAS or combination (BMP2+JAS) thereof and sealed without trapping any air bubbles. A number of randomly selected samples were cultured on a clinostat to simulate microgravity for 48 h as previously described [33], and others were placed into the same incubator without simulated microgravity as a parallel control. All cell samples were subsequently examined for fluorescence intensity, actin immunofluorescence and ChIP analysis. In addition, media from samples were collected for ALP detection.

### Analysis of Fluorescence Intensity

**Figure 1:**
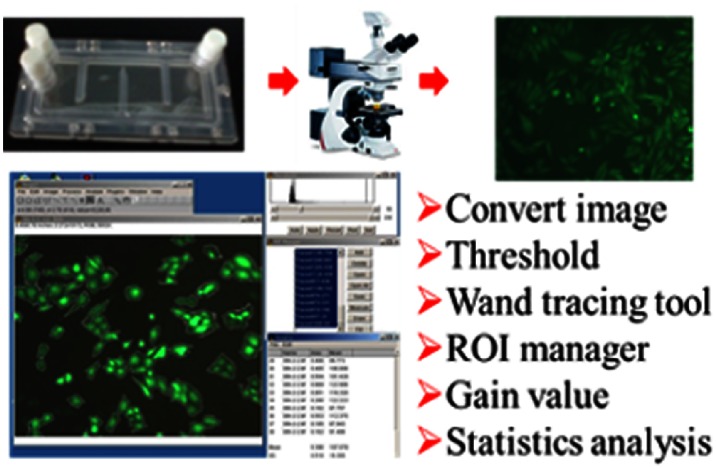
Schematic diagram of analysis of cell fluorescence intensity.

After treatment, cells were washed with PBS to reduce background. As described previously [33], fluorescence of cells was visualized using a fluorescence microscope with an H3 blue excitation filter system; images were captured with a Leica DC200 high resolution digital imaging system with the same setting parameters such as those for gamma value, exposure time and gain value and analyzed with the ImageJ software, a publically available Java-based image processing program developed by the National Institutes of Health [45]. In brief, the fluorescent image was converted into a 32-bit image, then used to analyze the intensity (FIi) and area value (Ai) of a selected single cell or cell regions as shown in Fig. 1. The minimal intensity of the whole image was counted as background intensity (FIb). The average fluorescence intensity (FI) of the image was calculated using the formula:
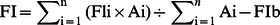



### Actin Staining

Cells were fixed with 4% paraformaldehyde for 30 min and preincubated in blocking buffer (1% BSA/PBS) for 30 min. They were then incubated in Texas red isothiocyanate conjugated phalloidin (Molecular Probes, USA) in blocking buffer at room temperature for 2 h, brieﬂy washed three times in PBS, and mounted in Mowiol. The stained cells were imaged by confocal microscopy.

### Chromatin Immunoprecipitation

OSE-MG63 cells were treated with BMP2 (200 ng/ml), CB (2.0 µmol/L), or combination thereof for 48 h. Following this, a ChIP analysis was performed according to manufacturer’s instructions using the ChIP assay kit from Beyotime (Nantong, China) and the Cbfa1 antibody from Santa Cruz. In brief, DNA and proteins were cross-linked by the addition of formaldehyde (1% final concentration) 10 min before harvesting, and cross-linking was terminated by the addition of glycine solution for 5 min at room temperature. Cells were scraped off the plates, resuspended in PBS with 1 mmol/L PMSF, collected by centrifugation, and lysed in SDS Lysis Buffer containing 1 mmol/L PMSF. The cell lysate was sonicated to generate 500–2000 bp fragments. After centrifugation, the supernatant was diluted 10-fold with ChIP Dilution Buffer and precleared by incubating at 4°C with Protein A+G beads preadsorbed with salmon sperm DNA. The cleared lysates were incubated overnight with Cbfa1 antibody. Immune complexes were precipitated with protein A+G beads. After centrifugation, the beads were washed and the antigen eluted with 1% SDS containing 100 mmol/L sodium carbonate. DNA-protein cross-links were added with NaCl (0.2 mol/L final concentration) and incubated at 65°C for 4 h. DNA was extracted using a DNA extraction kit (CoWin Biotech, Beijing, China) and qPCR was performed with the following primers using the SYBR Premix Ex Taq II kit (Takara, Dalian, China): 6OSE-EGFP-F: 5-GATCTCCAAC CACACCAACC-3, 6OSE-EGFP-R:5-GATCCGACTTGTCTGTTCTGC-3. OCN-F: 5-CGG GCAGTCTGATTGTGGC-3(-262), OCN-R: 5-GCCTCC AGCACTGTTTATACCC-3(-39).

### RT-qPCR

After treatment, total RNA was purified from harvested cells using Trizol reagent. 1 µg of total RNA was reversely transcribed into cDNA using PrimeScrip RT reagent kit with gDNA Eraser (Takara, Dalian, China) in a 20 µl reaction volume according to the manuscriptor’s instructions, then 1 µl of cDNA was used for qPCR with EGFP and GAPDH primers using the SYBR Premix Ex Taq II kit (Takara, Dalian, China). Relative expression levels of each gene were normalized to GAPDH RNA levels. EGFP-F 5-ACGTAAACG GCCACAA GTTC and EGFP-R 5-AAGTCGTGCTGCTTCATGTG; Cbfa1-F 5-CGCATTC CTCATCCCAGTAT and Cbfa1-R 5-TGGCTCAGATAGGAGGGGTA; GAPDH-F: 5-GTC TTCACCACCATGGAGAAGG and GAPDH-R: 5- GCCTGCTTCACCACCTTCTTGA. The relative expression data were analyzed using 2^−ΔΔCt^ methods.

### Western-blot Analysis

Total proteins of MG63 were extracted after treatment with BMP2, CB or both using a standard method and quantified using the BCA protein assay kit (thermo Scientific, Pierce, USA). Whole cell protein extracts (40 µg/lane) were separated by SDS-PAGE and transferred to a PVDF membrane(Millipore, USA). The membrane with proteins was blocked in TBST containing 5% nonfat milk for 0.5 h at RT, followed by overnight incubation at 4°C with primary antibodies against Smad1, Phospho-Smad1/5/8 (all from Cell Signaling, catalog, no. 9743, 9511L; USA) at dilutions of 1∶200 with blocking buffer. Blots were washed (three times) with TBST the following day for 30 min and incubated with the appropriate peroxidase-conjugated second antibodies for 1 h. Bound antibodies were detected with enhanced chemiluminescence (Thermo Scientific, Pierce, USA).

### Statistical Analyses

All data were expressed in mean ± standard deviation (SD). Data among more than three groups were analyzed by one-way ANOVA followed by Bonferroni’s multiple comparison using SPSS 18 program and data between two groups were analyzed by t–test. p<0.05 was considered to represent a significant difference.

## Results

### Construction Osteoblast Reporter Line for Cbfa1 Activity

We constructed an osteoblast reporter cell line using the cis-acting element of mouse osteocalcin gene 2 (OSE2) as a readout of Cbfa1 activity. The reporter p6OSE2-EGFP contains 6 OSE2 elements upstream of a minimal 34 bp mOG2 promoter (Ducy and Karsenty 1995), and the enhanced green fluorescence protein (EGFP) reporter. The vector was sequenced to confirm the promoter sequence (Fig. S1). MG63 cells were transfected with p6OSE2-EGFP and stably transfected cells were selected with the antibiotic G418. Six clones with high fluorescence were obtained for the second round of selection by limiting dilutions in G418 selection medium. One of these clones is shown in Fig. S2. The osteoblast reporter cell line established here was named 6OSE2-EGFP-MG63 and abbreviated as OSE-MG63.

### Responsiveness of the OSE-MG63 Cell Line to Cytokines

**Figure 2:**
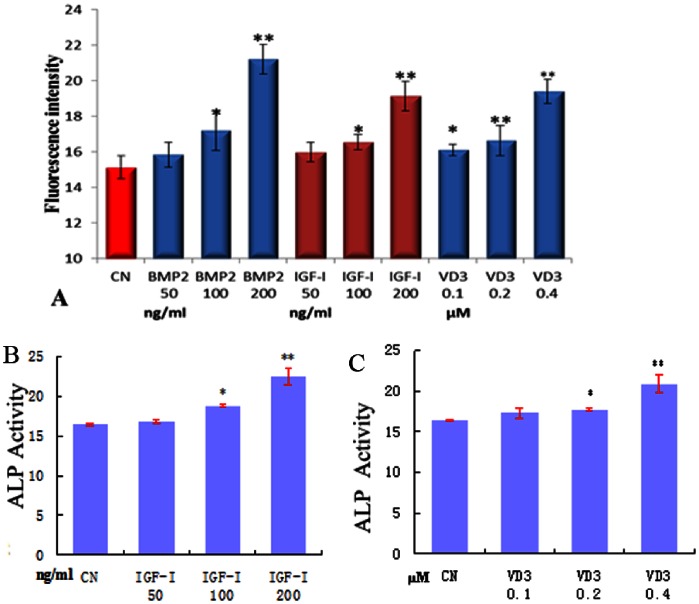
Responsiveness of the OSE-MG63 cell line to BMP2, IGF-I and VD3. OSE-MG63 cells were treated with different concentrations of these cytokines for 48 h, after which fluorescence intensity (A) and ALP activity (B, C) were analyzed. * P<0.05, ** P<0.01, vs. CN (untreated), n = 3.

To test the responsiveness of the OSE2 regulatory factor in the reporter cell line, OSE-MG63 cells were treated with different concentrations of IGF-I, VD3 or BMP2, and reporter fluorescence was analyzed 48 h later. As shown in Fig. 2A and Fig. S3, fluorescence gradually increased with higher concentrations of IGF-I, VD3 or BMP2, and was strongest at 200 ng/ml IGF-I, 200 ng/ml BMP2 and 0.4 µmol/L VD3 among tested concentration. An increase in ALP activity, as shown in Fig. 2B, C and Fig. S3, demonstrates the increase of osteogenic differentiation following the addition of higher concentrations of IGF-I and VD3. The parallel increase in fluorescence intensity and ALP activity upon increasing concentrations of factors suggests that the reporter fluorescence reflects Cbfa1 activity and osteogenic differentiation. We selected 200 ng/ml as the optimal concentration for BMP2 exposure in subsequent experiments.

### Effects of Microgravity on Cbfa1 Activity and Responsiveness to BMP2

**Figure 3:**
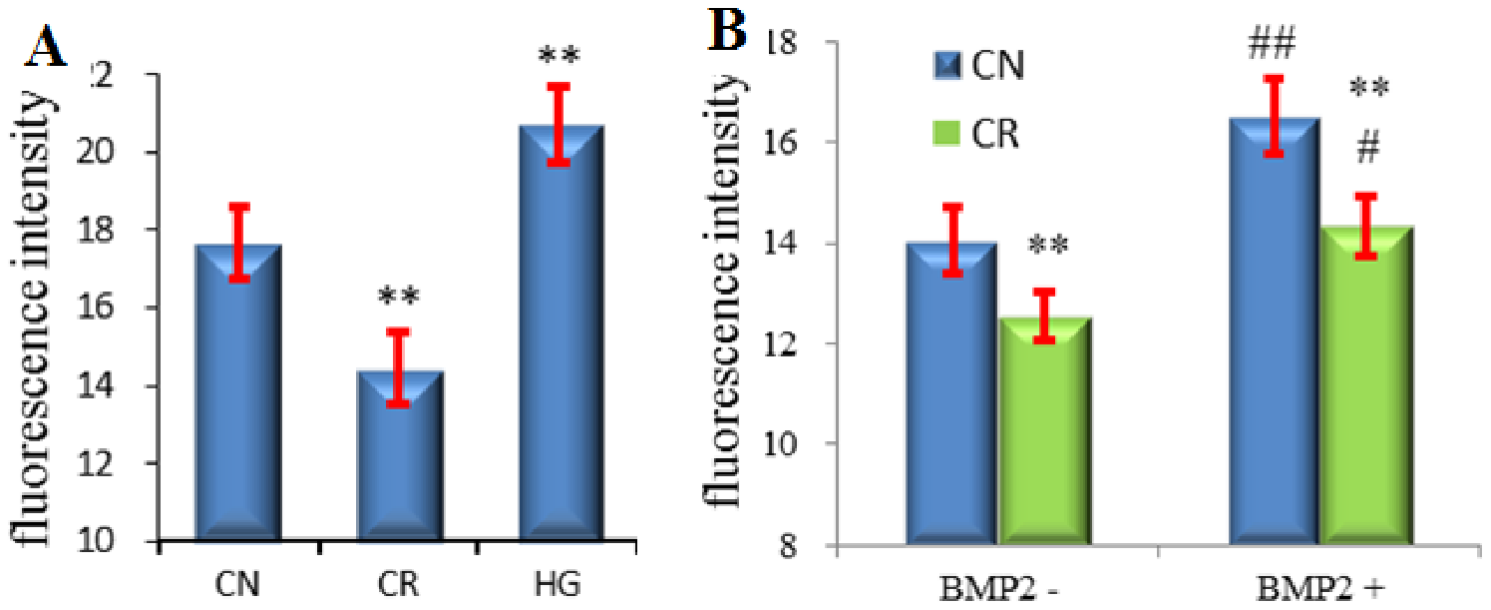
Effects of microgravity and hypergravity on Cbfa1 activity and responsiveness to BMP2. OSE-MG63 cells were cultured in a clinostat or in a cell centrifuge for 48 h with or without BMP2 (200 ng/ml), after which the fluorescence intensity was analyzed. CN: normal gravity, CR: clinorotation, HG: hypergravity ** P<0.01, vs. CN, # P<0.05, ## P<0.01 vs. BMP2−, n = 3.

It is well known that microgravity influences the expression of Cbfa1 and inhibits osteogenesis. Here we investigated the effects of microgravity and hypergravity on Cbfa1 activity using OSE-MG63 reporter line. OSE-MG63 cells were cultured in normal gravity (Control, CN), simulated microgravity (clinorotation, CR) (10^−3^∼^−2^ g) and hypergravity (cell centrifugation, HG) (3 g) for 48 h. Compared with normal gravity culture, simulated microgravity caused a significant decrease and hypergravity a marked increase in reporter fluorescence intensity (Fig. 3A). The OSE-MG63 cell line displayed the same decreased reporter fluorescence intensity in response to microgravity on ShenZhou-7 spaceflight mission (data not shown). Addition of 200 ng/ml BMP2 in microgravity conditions increased reporter fluorescence intensity, but simulated microgravity markedly attenuated the promotive effects of BMP2 on Cbfa1 compared to normal gravity culture (Fig. 3B). The increased effect of BMP2 on fluorescence intensity is 19.73% ((16.81–14.04)/14.04) under normal gravity and is 14.19% ((12.54–14.32)/12.54) under simulated microgravity. From these results, we conclude that microgravity inhibited the responsiveness of Cbfa1 to BMP2.

### Microfilament Participates in BMP2-induced Cbfa1 Activity

**Figure 4:**
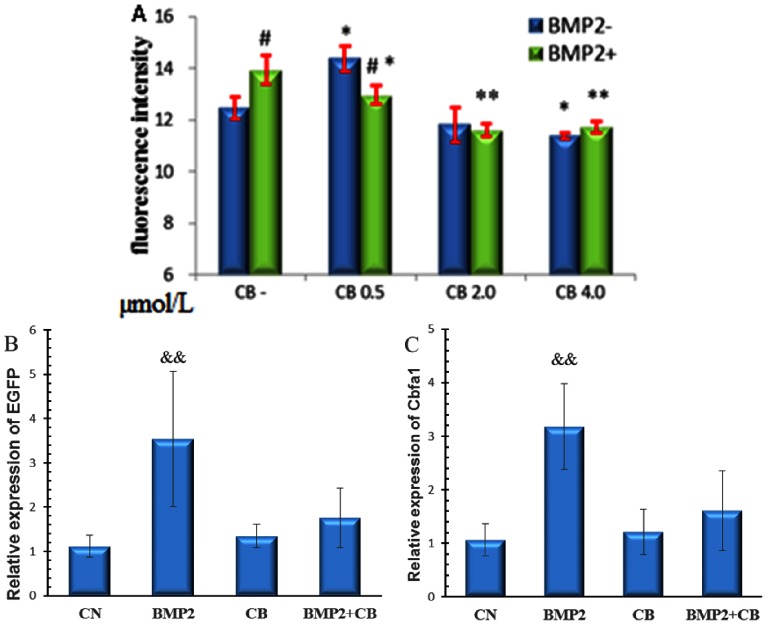
Effects of CB on Cbfa1 activity and BMP2 induction effect. OSE-MG63 cells were treated with different concentrations of CB with or without 200 ng/ml BMP2 for 48 h, then fluorescence intensity (A) and mRNA level of EGFP (B) and Cbfa1 (C) was analyzed. The CB concentration is 2.0 µmol/L in mRNA analysis (B, C). && p<0.01, VS. other groups n = 5.

Gamell and colleagues demonstrated that BMP2 induces a rapid actin cytoskeletal rearrangement in pluripotent C2C12 cells [25]. We hypothesized that the actin cytoskeleton is important for BMP2 induction to Cbfa1 activity. Cytochalasin B (CB), which disrupts F-actin formation, was added with or without 200 ng/ml BMP2 at different concentrations (0.5, 2.0, 4.0 µmol/L) for 48 h. As shown in Fig. 4A, reporter fluorescence intensity increased following treatment of low (0.5 µmol/L) concentrations and decreased following higher concentrations (2.0, 4.0 µmol/L) of CB. A particularly marked decrease was observed with 4.0 µmol/L CB when compared with the control group (CB−+BMP2−). In BMP2 treatment groups, low concentrations of CB (0.5 µmol/L) were sufficient to eliminate the stimulation of reporter fluorescence and higher CB concentrations further decreased the fluorescence intensity significantly. However, we observed no difference in inhibitory effects between 2.0 µmol/L and 4.0 µmol/L CB treatment groups. The mRNA level of reporter EGFP and Cbfa1 were also analyzed by RT-qPCR after treatment with BMP2 (200 ng/ml) and/or CB (2.0 µmol/L) for 48 h. As shown in Fig. 4BC, BMP2 significantly increased the expression of EGFP and Cbfa1, but not when combined with CB treatment. Lower expression of EGFP and Cbfa1 was detected, likely due to a decrease in the inductive influence of BMP2 by CB. These results were coincided with the reporter fluorescence intensity analysis. These data suggest that intact microfilament networks are important for BMP2 driven osteogenesis.

**Figure 5:**
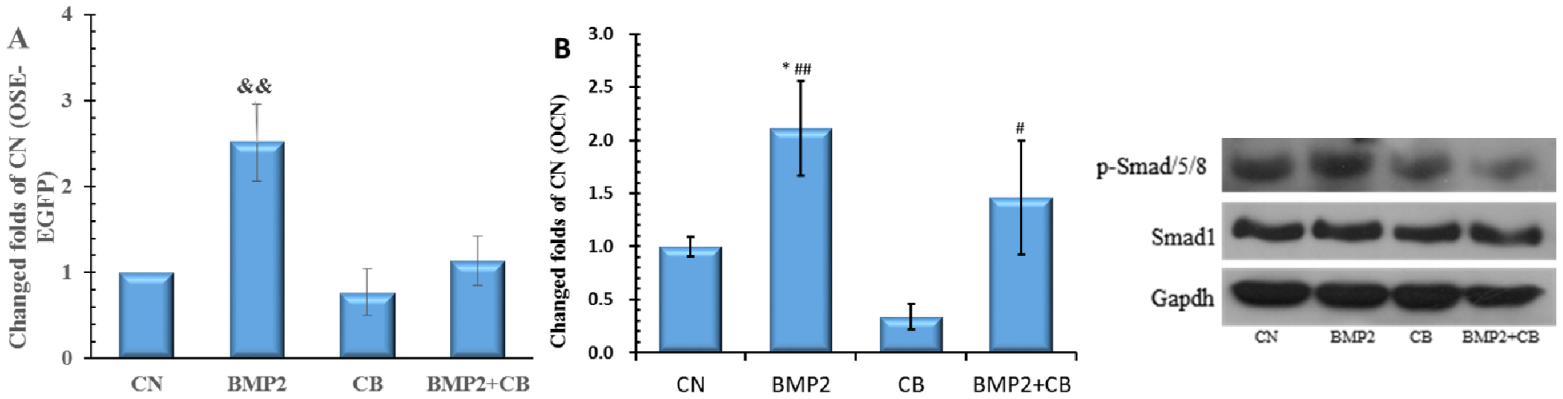
CB suppresses DNA binding activity of BMP2-induced Cbfa1 and phosphorylated level of Smad1/5/8. OSE-MG63 cells were treated with BMP2 (200 ng/ml), CB (2.0 µmol/L) or a combination thereof for 48 h, then analyzed by ChIP. The chromatin fragment of 6OSE2 (A) or osteocalcin (B) promoter immunoprecipitated by the Cbfa1 antibody was assayed by quantitative PCR and expressed as a relative value against the amount of input DNA. The values represent the averages plus standard errors (error bars) from triplicate samples. && p<0.01 vs. other groups * p<0.05, VS. CN, #p<0.05, ## p<0.01 VS. CB, n = 3. (C) Proteins were analyzed for phosphorylation of Smad1/5/8 by Western blot. Total SMAD1 and GAPDH protein level showed equal loading of protein (representative of n = 3).

To further characterize the involvement of F-actin in BMP2 induction of Cbfa1, binding activity of Cbfa1 to OSE2 was studied using a ChIP assay method. OSE-MG63 cells were treated with BMP2 (200 ng/ml), CB (2.0 µmol/L), or both for 48 h, then analyzed by ChIP. Quantitative PCR analysis of immunoprecipitated DNA revealed that chromatin fragments containing Cbfa1 binding sites (6OSE2) pull downed by Cbfa1 antibody significantly increased in the BMP2 treatment group and almost no change was observed in the presence of CB (CB and CB+BMP2 groups) as shown in Fig. 5A. The same changes were observed using the primers of osteocalcin promoter, which contains the Cbfa1 binding site (Fig. 5B).

To explore the effects of microfilament on BMP2 signaling, the MG63 cells were treated with BMP2 (200 ng/ml), CB (2.0 µmol/L), or both for 24 h, then analyzed by western blot for the phospho-Smad1/5/8. As shown in fig. 5C, BMP2 increases the phosphorylated Smad1/5/8 level, but addition of CB could inhibited the phosphorylated Smad1/5/8 level. These results suggest that microfilament network participates the BMP2 signaling.

### Simulated Microgravity Disrupts Microfilament Organization in MG63 Cells

**Figure 6:**
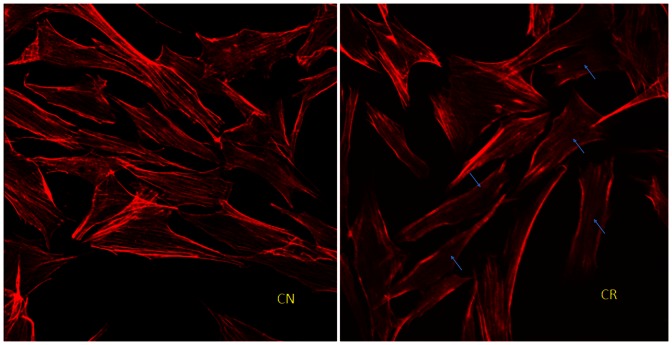
Simulated microgravity disrupts F-actin in MG63 cell line. Cells were cultured in clinorotation for 48 h and stained with Texas red isothiocyanate-conjugated phalloidin. CR: clinorotation, CN: control.

The cytoskeletal system is sensitive to microgravity. After culturing in simulated microgravity condition (clinostat) for 48 h, actin filaments of MG63 cells depolymerized, became thinner, and showed a dispersed distribution and disorder, especially in the cytoplasm (Fig. 6, with blue arrow). This is consistent with previous observations by ourselves and other research teams using other cell lines [33], [46].

### JAS Reverses Inhibition by Microgravity of BMP2-induced Cbfa1 Activity

**Figure 7:**
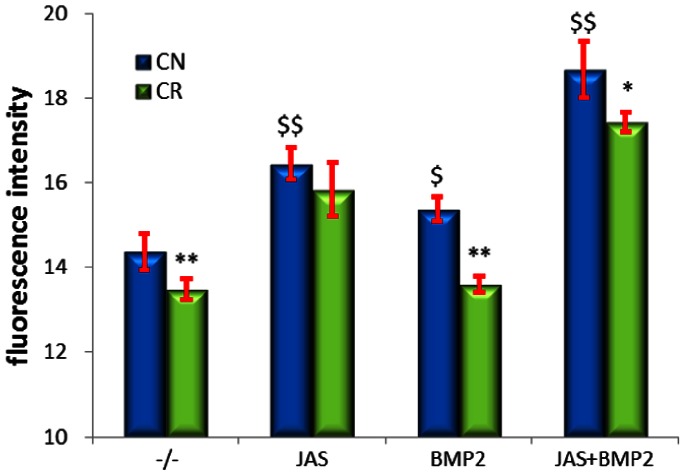
Effects of JAS and BMP2 on the changes of Cbfa1 activity induced by clinorotation. OSE-MG63 cells were treated with JAS, BMP2 or a combination thereof, and cultured in clinostat for 48 h, after which the fluorescence intensity was analyzed $P<0.05, $$ P<0.01, VS. −/−(untreated group), * P<0.05, ** P<0.01 vs. CN. n = 4.

JAS, an actin polymerizing and microfilament stabilizing drug, was added with BMP2 during culture of OSE-MG63 cells in simulated microgravity (clinorotation) to examine its protective function. In normal gravity, treatment with JAS, BMP2, or both increased reporter fluorescence dramatically (Fig. 7). Although reporter fluorescence after JAS treatment appeared lower in simulated microgravity, there was no statistically significant discrepancy (p = 1.00) between normal gravity and simulated microgravity. In the BMP2 treatment group, the fluorescence intensity significantly decreased 11.4% in simulated microgravity (CR+BMP2) compared with normal gravity (CN+BMP2), and only decreased 6.67% after addition of JAS in simulated microgravity (CR+BMP2+JAS) compared with normal gravity condition (CN+BMP2+JAS). Furthermore, JAS and BMP2 had an accumulative effect on the fluorescence intensity levels, because higher reporter fluorescence was observed in the JAS+BMP2 group compared with individual treatments of JAS or BMP2 in normal gravity or simulated microgravity. The above results suggest that JAS confers some protective function from the inhibitory effect of simulated microgravity on the induction of osteogenesis by BMP2.

## Discussion

Accumulating evidence demonstrates that microgravity inhibits the initial as well as subsequent stages of osteoblast differentiation [6], [47]. Cbfa1, an osteoblast-specific transcription factor, not only initiates the differentiation of osteoblasts, but regulates the expression of osteoblast-specific genes during differentiation. Expression of Cbfa1 is decreased under real and simulated microgravity [48]. On the other hand, we and other investigators have demonstrated that microgravity decreases the responsiveness of osteoblasts to cytokines that promote osteoblast proliferation, differentiation and bone formation, such as BMP2 and IGF-I. These cytokines regulate the expression and activity of Cbfa1 during osteogenesis [9], [20]. The mechanisms by which microgravity has an effect on Cbfa1 activation or inhibits its responsiveness to cytokines are not clearly understood. Here, we investigated whether the effects of microgravity on BMP2-induced osteogenic differentiation are related to actin disruption.

We first engineered an osteoblastic reporter cell line with which Cbfa1 activity alternation can be directly monitored by fluorescence intensity. The human osteosarcoma cell line MG63 is an undifferentiated osteoblast-like cell line and expresses osteoblast markers such as collagen type I, ALP, and osteocalcin whose expression is enhanced by VD3 [49]. Cbfa1 modifies the transcription of osteoblastic genes by binding to its response element (OSE2) in the promoter of target genes [43], [50]. Since this OSE2 element plays a crucial role in inducing and controlling bone related gene expression [51], [52], an artificial promoter composed of six tandem copies of OSE2 sequence and a minimal mOG2 (mouse Osteocalcin Gene 2) promoter [43] has been widely used to study Cbfa1 activity [53], [54]. In present, we created the reporter cell line OSE-MG63, containing a stably transfected reporter vector consisting of the 6OSE2 promoter upstream of EGFP reporter. The increase of reporter fluorescence upon treatment with increasing concentrations of IGF-I and VD3 mirrors the change in ALP activity in response to these factors [55], [56]. These data suggest that the fluorescence levels reflect the activity of Cbfa1 and that the OSE-MG63 cell line can be used to study the mechanisms underlying the effect of microgravity on Cbfa1 activity.

Next, to confirm the suitability of the reporter cell line OSE-MG63 in our studies, we examined the effects of microgravity and hypergravity on reporter activity. The expression of Cbfa1 decreases under microgravity, but increases under hypergravity conditions [57]. We have also demonstrated that overexpress Cbfa1 by transfection with exogenous gene into MC3T3-E1 only partly antagonize the decreased expression of osteogenic specific molecules induced by simulated microgravity, which suggest that microgravity affects the activity of Cbfa1, but not only its expression [58]. In present, our results, especially in spaceflight results (data not shown), using OSE2-driven EGFP expression as a reporter for Cbfa1 activity were consistent with this, Which confirm the reporter cell line OSE-MG63 as an applicable model for exploring the mechanisms of microgravity on Cbfa1 activity. In a previous study we demonstrated that simulated microgravity decreases the promotive effects of IGF-I on the proliferation of bone marrow mesenchymal stem cells [9]. Microgravity reduces the differentiation of osteoblastic MG63 cells in response to VD3 and TGFβ2 [59]. BMP2, one of the most potent osteoblastic inducers, is known to control the activity and expression of Cbfa1 by Smad signaling and to regulate bone-related genes by Cbfa1, suggesting that the BMP2-Cbfa1 axis plays important roles in osteogenesis [14], [60]–[62]. Simulated microgravity by a random positioning machine prevented mineralization of 2T3 preosteoblasts induced by BMP2 or BMP4 [63]. Moreover, BMP2 treatment increases serum corticosterone and indirectly attenuates GFAP mRNA in the stratum molecular of the hippocampus in normal gravity, but not in microgravity [64]. In the present study, fluorescence intensity of the reporter cell line OSE-MG63 significantly increased after BMP2 treatment in normal gravity, but only reached control levels under simulated microgravity conditions. These results combined with previous reports suggest that microgravity modulates osteoblast Cbfa1 responsiveness to BMP2.

Using our Cbfa1 reporter line, we next investigated our hypothesis that disrupted actin microfilament plays a vital role in the decreased responsiveness of Cbfa1 activity to BMP2. It is well known that the actin cytoskeleton network is sensitive to altered gravity and its depolymerization, extenuation and dispersed distribution has been observed in different cell lines exposed to altered gravity [9], [65]–[67]. Our previous work demonstrated that actin microfilament participates in the regulation of activity at the COL1A1 promoter in ROS17/2.8 cells under simulated microgravity [33]. In addition, BMP2 can induce a rapid, significant and transient rearrangement of the dynamic actin cytoskeleton in C2C12 cells [25]. Whether the affected actin cytoskeleton in turn attenuates the effect of BMP2 is not well known. An intact, dynamic actin cytoskeleton network under tension is necessary for oscillatory fluid flow-induced gene expression such as Cbfa1 [68]. BMP-2-induced ALP activity was decreased by actin-binding protein CNh1 expression [69]. In the present study (Fig. 4), actin disruption by CB at a low concentration decreased the reporter fluorescence and mRNA expression in OSE-MG63 cells treated with BMP2, which suggests that actin microfilament takes part in promotive effects of BMP2 on osteoblast differentiation. A higher concentration of CB (2.0 µmol/L) almost eliminated the effect of BMP2 on Cbfa1 activity and mRNA level of EGFP and Cbfa1. Actin disruption by CB (0.5 µmol/L) or low JAS increases the activity of Cbfa1, but decreases it at higher CB concentrations. These results suggest that the integrity of actin cytoskeleton network is necessary for BMP2 function in osteoblast differentiation.

Finally, we asked whether actin disruption induced by microgravity plays an important role in the inhibitory effect of microgravity on BMP2 induction of Cbfa1 activity. When OSE-MG63 cells were cultured in simulated microgravity conditions, BMP2’s induction to Cbfa1 activity was significantly compromised. However, addition of F-actin polymerizing and stabilizing drug JAS decreased microgravity’s inhibition on BMP2-induced Cbfa1 activity. In normal gravity, JAS and BMP2 have a cumulative effect on the fluorescence intensity, indicating that JAS confers some protective function from the inhibition of simulated microgravity on the induction of osteogenesis by BMP2.

### Conclusion

Taken together, we conclude that 1) Cbfa1 activity and osteogenesis is mirrored by fluorescence intensity of EGFP in the OSE-MG63 reporter cell line; 2) simulated microgravity inhibits Cbfa1 activity and its responsiveness to BMP2; and 3) actin microfilament participates in BMP2’s induction to Cbfa1 activity and their disruption might contribute to the inhibition of BMP2’s osteogenic functions under simulated microgravity. A sketch has been draw about this point in Figure S4.

## Supporting Information

Figure S1 **Sequence of 6OSE2 promoter in the p6OSE2-EGFP expression vector.** The uppercase is the sequence of 6OSE and the lowercase is the sequence from pEGFP-N1.
(TIF)

Figure S2 **Fluorescence and bright images of an OSE-MG63 clone.**
(TIF)

Figure S3 **Fluorescence (top) and ALP staining (bottom) images of OSE-MG63 cells treated with IGF-I for 48 h (A control; B 50 ng/ml; C 100 ng/ml; D 200 ng/ml).** After treatment, fluorescence images were taken before performing ALP staining using the modified calcium and cobalt method.
(TIF)

Figure S4 **Model sketch for microfilament network takes part in the BMP2 induction to Cbfa1 activity which was described in present study.**
(TIF)
