# Supplementary figures and images for: Actin Microfilament Mediates Osteoblast Cbfa1 Responsiveness to BMP2 under Simulated Microgravity
Source: PLoS One. 2013 May 10;8(5):e63661. doi: 10.1371/journal.pone.0063661 (PMC3651164; doi:10.1371/journal.pone.0063661)

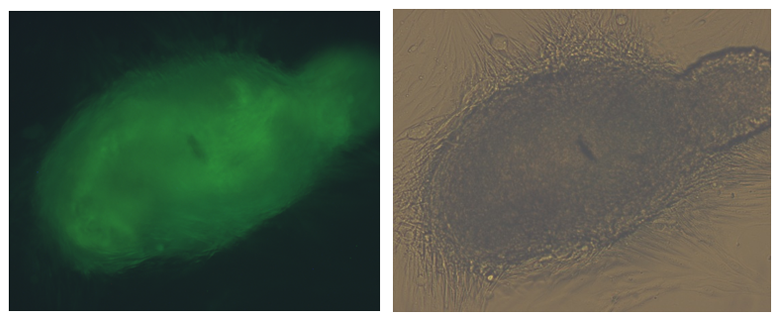

Supplement: Figure S2 — Fluorescence and bright images of an OSE-MG63 clone. (TIF) [file pone.0063661.s002.tif]

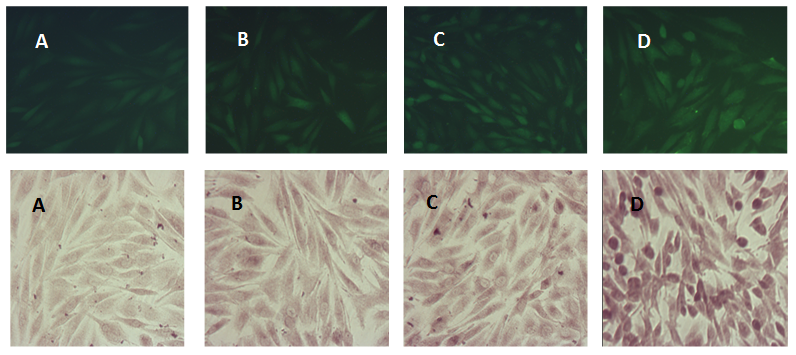

Supplement: Figure S3 — Fluorescence (top) and ALP staining (bottom) images of OSE-MG63 cells treated with IGF-I for 48 h (A control; B 50 ng/ml; C 100 ng/ml; D 200 ng/ml). After treatment, fluorescence images were taken before performing ALP staining using the modified calcium and cobalt method. (TIF) [file pone.0063661.s003.tif]

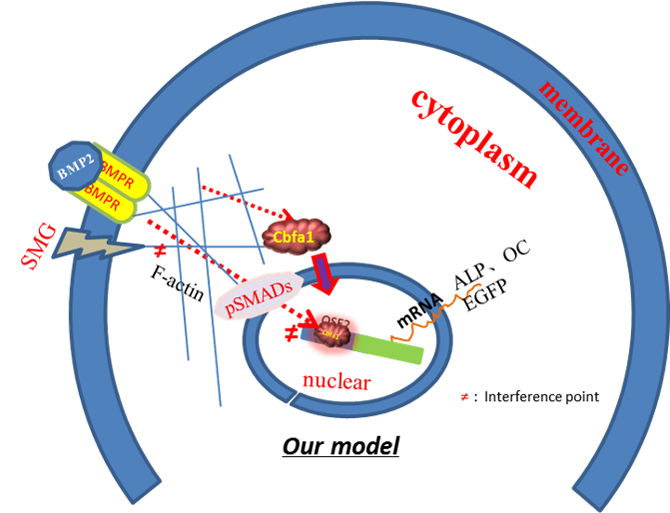

Supplement: Figure S4 — Model sketch for microfilament network takes part in the BMP2 induction to Cbfa1 activity which was described in present study. (TIF) [file pone.0063661.s004.tif]
